# Psychometric properties of the Chinese Mandarin version of the Borderline Symptom List, short form (BSL-23) in suicidal adolescents

**DOI:** 10.1186/s40479-023-00230-3

**Published:** 2023-08-09

**Authors:** Jui-En Shen, Yu-Hsin Huang, Hui-Chun Huang, Hui-Ching Liu, Tsung-Han Lee, Fang-Ju Sun, Chiu-Ron Huang, Shen-Ing Liu

**Affiliations:** 1 Department of Psychiatry, MacKay Memorial Hospital, Taipei, Taiwan; 2 Department of Medicine, MacKay Medical College, New Taipei City, Taiwan; 3 Department of Medical Research, MacKay Memorial Hospital, Taipei, Taiwan; 4 MacKay Junior College of Medicine, Nursing and Management, Taipei, Taiwan

**Keywords:** Borderline personality disorder, Borderline Symptom List, Adolescents, Instrumental study, ROC curve

## Abstract

**Background:**

The short form of the Borderline Symptom List (BSL-23) is a self-rated instrument developed from the initial 95-item German version of the Borderline Symptom List (BSL-95). It is widely used among Chinese adults, but its applicability, factor structure and validity remain uncertain in adolescents. This study aimed to evaluate the psychometric properties of the Chinese Mandarin version of the BSL-23 in a sample of suicidal adolescents.

**Methods:**

The Chinese Mandarin BSL-23 was given to 279 outpatient adolescents with self-injurious thoughts or behaviors. The factor structure, reliability, convergent validity, criterion-related validity and cut-off value were investigated.

**Results:**

The Chinese Mandarin version of the BSL-23 demonstrated a one-factor structure and replicated the original version. The scale had high reliability and good test-retest stability. The Chinese Mandarin BSL-23 was correlated with depression, hopelessness, impulsivity, emotional dysregulation, self-esteem, loneliness, childhood trauma and parental bonding patterns evaluated with a variety of scales. The measure showed good criterion-related validity and predictive accuracy (AUC = 0.87) for self-injurious and suicidal adolescents with borderline personality disorder (BPD) at a cut-off point of 60/61 (mean score 2.60/2.65), with a sensitivity of 0.76 and specificity of 0.83.

**Conclusions:**

The Chinese Mandarin version of the BSL-23 is a reliable and valid self-reported instrument to assess BPD symptomatology among suicidal adolescents.

## Introduction

Borderline personality disorder (BPD) is a common and severe mental disorder, characterized by emotional dysregulation, impulsivity, self-damaging and suicidal behaviors, identity disturbance, and low self-esteem. BPD is usually associated with other psychiatric and personality disorders, severe functional impairment, high burden on families, poor socio-economic and familial outcomes, continuing resource utilization, and a high suicide rate. A syndrome of BPD typically onsets during adolescence and can be distinguished reliably from normal adolescent development [1]. The fifth edition of the Diagnostic and Statistical Manual of Mental Disorders (DSM-5) permits the diagnosis of BPD in patients younger than 18 years if symptoms persist for at least 1 year. Recent evidence has demonstrated that BPD is as reliable and valid among adolescents as it is in adults. Adolescent BPD is frequently characterized by an over representation of risk-taking and self-harming behaviors. Over 30% of patients with BPD have been reported to begin self-harming when aged 12 years or less, with another 30% initiating self-harm between the ages of 13 and 17 [2].

Several studies have reported that the prevalence rate of BPD in adolescents is similar to that in adults, with 1–3% in community samples, 33–49% in clinical samples, and 11% in outpatient samples [3]. A wide range of risk factors in childhood have been identified, including individual (such as depression, anxiety, dissociation, suicidality, self-injury, impulsive-aggressive behavior, attention deficit hyperactivity disorder and substance use disorder) and parental factors (such as low social economic status, family adversity, maternal psychopathology, exposure to physical or sexual abuse or neglect and specific parenting practices such as harsh punishment, invalidation, over-involvement, overprotection, lack of care, conflictual and inconsistent relationships, emotional withdrawal or unavailability and role reversal) [3–5].

Borderline pathology prior to the age of 19 years has been shown to be predictive of long-term deficits in functioning [6]. Adolescents with BPD can benefit from early detection and intervention, and the diagnosis and treatment of BPD should be considered part of routine practice in adolescent mental health to improve their well-being.

Multiple structured, semi-structured and self-rated instruments have been developed and validated to evaluate borderline symptomatology among adolescents with BPD. The psychometric properties of some self-reported questionnaires have been investigated for BPD among Chinese adolescents, including the McLean Screening Instrument for BPD, Personality Diagnostic Questionnaire-4+, and Borderline Personality Features Scale for Children [7–9]. However, no instrument has been validated or cut-off values identified for high-risk adolescents in the community, and the present study aimed to fill this gap.

The initial Borderline Symptom List (BSL) included 95 items based on DSM-IV, the Diagnostic Interview for Borderlines-Revised (DIB-R), and the opinions of clinicians and BPD patients. In the BSL-95, the intensity of each frequently made complaint (such as “I thought of hurting myself” or “I was lonely”) is evaluated over the previous week by the patients on a 5-point Likert scale, ranging from 0 (none) to 4 (very strong). The BSL-95 has been shown to have good psychometric properties, but its length makes it time consuming. Consequently, a shorter version, the BSL-23, was developed by Bohus et al. The BSL-23 consists of 23 items and has been shown to have high correlation with the BSL-95, high internal consistency, and high validity for discriminating patients with BPD from those with a DSM-IV axis I diagnosis, such as post-traumatic stress disorder and attention-deficit hyperactivity disorder [10]. In addition, principal component analysis has suggested a one-factor structure. The BSL-23 has been shown to be sensitive to symptom change following dialectical behavior therapy (DBT) [10], and to possess good psychometric properties in several languages [11–13]. Correlations between the BSL-23 and several instruments used to assess BPD psychopathology, such as lower self-esteem [14], childhood trauma [15] and emotional regulation [16], have been explored in adults. However, such correlations have not been investigated among adolescents.

The aim of this study was to examine the psychometric properties of the Chinese Mandarin version of the BSL-23 in adolescents with self-injurious thoughts or behaviors. Internal consistency, factor structure, test-retest reliability and area under the curve (AUC) were explored. Correlations between the Chinese Mandarin version of the BSL-23 and other psychiatric scales were assessed. Furthermore, its validity to discriminate BPD patients from those without BPD in high-risk adolescents was also investigated.

## Methods

### Participants

Adolescents aged 12–18 years who were involved in self-injurious thoughts or behaviors (SITB) were recruited from the psychiatric outpatient clinics of Mackay Memorial Hospital (MMH), a medical center in Taipei, Taiwan between October 2018 and December 2021 if they responded “yes” to any of the screening questions: “Have you ever thought about killing yourself?”, “Have you ever made an actual attempt to kill yourself in which you had at least some intent to die?” and “Have you ever actually purposely hurt yourself without wanting to die?” All parents provided written consent and the adolescents provided written assent. They were then interviewed using the following instruments: (1) Kiddie Schedule for Affective Disorder and Schizophrenia-Epidemiological version for School-Age Children (K-SADS-E); (2) Self-Injurious Thoughts and Behaviors Interview (SITBI), and (3) Structured Clinical Interview for DSM-IV axis II Personality Disorders (SCID-II). The adolescents completed the self-reported measures. Additional information was provided by their parents or legal guardians. The adolescents were excluded from the study if they were unable to complete the protocol due to schizophrenia spectrum and other psychotic disorders, cognitive deficits, or extreme suicide-related behaviors. Each of the adolescents and parents was compensated with 250 New Taiwan Dollars (around 8 US Dollars) for the interviews. This study was approved by the Institutional Review Boards of MMH.

## Instruments

### Diagnostic interview

#### Self-Injurious Thoughts and Behaviors Interview (SITBI)

The SITBI is a structured interview that quantifies the presence, frequency and severity of nonsuicidal self-injury as well as suicidal thoughts and behaviors [17]. The SITBI has demonstrated good reliability and validity among adolescent community patients [18]. The Chinese version of the SITBI has been translated and back-translated, and it has been shown to be valid and reliable [19].

#### BPD Subscale (Chinese version) of the Structured Clinical Interview for DSM-IV Axis II Personality Disorders (SCID-II)

The BPD subscale (Chinese version) of the SCID-II was evaluated by a child psychiatrist. Good internal consistency, diagnosis agreement, sensitivity, and specificity have been demonstrated [20]. The diagnosis of BPD was made according to the BPD subscale of the SCID-II.

### Self-reported scales

#### Borderline Symptom List, short form (BSL-23)

The BSL-23 is a self-rated questionnaire that measures the severity of BPD symptomatology [10]. Each item is scored on a 5-point Likert scale, ranging from 0 (none) to 4 (very strong). Higher scores indicate higher BPD severity [12]. It has been translated into many languages [21] and shown good psychometric properties among adults in Spanish, French and Simplified Chinese [11–13]. It was translated into Chinese by an independent translator and back-translated to English. It has been implemented in BPD patients receiving DBT in Taiwan, and it has been shown to be sensitive to changes in suicidal ideation and behaviors [22].

#### Beck Hopelessness Scale (BHS)

The BHS is a 20-item true/false self-reported questionnaire used to assess negative attitudes about the future, with higher scores indicating greater hopelessness [23]. The BHS has been demonstrated to have strong psychometric properties in adolescents [24]. The Chinese version of the BHS has been shown to be reliable and valid in adults with self-harm behavior [25]. In this study, the Cronbach’s alpha was 0.902.

#### Barratt Impulsiveness Scale, 11th version (BIS-11)

The BIS-11 is a 30-item self-reported questionnaire designed to measure impulsivity, with higher scores indicating greater impulsivity [26]. The 25-item Chinese version of the BIS-11, in which five items were removed due to weak item–total correlation, has been reported to have good internal consistency in adolescents [27], and it has been used in adolescent studies in Taiwan [27, 28].

#### Childhood Trauma Questionnaire, short form (CTQ-SF)

The CTQ-SF [29] is a 25-item self-reported questionnaire used to assess the respondent’s experiences of childhood trauma. Item scores range from 1 (never true) to 5 (very often true). The questionnaire is composed of five subscales: emotional abuse, physical abuse, sexual abuse, emotional neglect, and physical neglect. The Chinese version of the CTQ-SF has been validated and used in adolescents [30].

#### Difficulties in Emotional Regulation Scale (DERS)

Emotional dysregulation was assessed with the DERS, in which participants indicate on a Likert scale ranging from 1 (almost never) to 5 (almost always) how often each item applies to themselves [31]. Higher scores indicate greater emotional dysregulation. Internal consistency and test-retest reliability have been reported to be good among Chinese adults and adolescents [32]. In this study, the Cronbach’s alpha was 0.930.

#### Parental Bonding Instrument (PBI)

The PBI [33] is a 25-item self-reported questionnaire in which children rate their parental care (affection and warmth vs. rejection and indifference), and parental authoritarian control over his or her behaviors and overprotectiveness (psychological autonomy vs. overprotection). Higher scores in the care and protection subscales indicate that the child perceives his or her parents to be more caring and/or protective. The Chinese version of the PBI has shown fair psychometric properties in Taiwanese young adults [34], and it has been widely used in Taiwanese studies [35].

#### Patient Health Questionnaire-9 item (PHQ-9)

The PHQ-9 consists of nine items, with higher scores indicating an increased likelihood of major depressive disorder. The Chinese version of the PHQ-9 has been shown to have good internal consistency and acceptable test-retest reliability among Taiwanese adolescents [36].

#### Rosenberg Self-Esteem Scale (RSES)

Self-esteem was assessed using the RSES. The RSES consists of 10 items, with higher scores indicating higher levels of self-esteem [37]. The reliability and validity of the Chinese version have been demonstrated in Taiwanese children and adolescents [38].

#### UCLA Loneliness Scale (UCLA-LS)

Loneliness was measured using a 10-item version of the UCLA-LS, with higher scores indicating a greater extent of loneliness [39]. The Chinese version has been validated among Chinese undergraduates [40]. In this study, the Cronbach’s alpha was 0.875.

### Statistical analysis

Data analysis was carried out using SPSS version 25 (IBM Corp., Armonk, NY), AMOS 24.0, and Python 3.1. Descriptive statistics were used to describe the demographic and clinical characteristics of the sample. To test the internal consistency, a global Cronbach’s alpha was estimated, and the split-half method was applied. Test-retest reliability was evaluated on a subsample of 32 participants over a one-week interval.

To measure the appropriateness of the factor analysis, the Kaiser-Meyer-Olkin measure and Bartlett’s test of sphericity were used. An exploratory factorial analysis of principal components with a varimax rotation was performed to examine the factorial structure. A confirmatory factor analysis was then performed to test the adequacy of the one-factor model. The accuracy of the fit was tested with chi-squares, standardized root mean square residual (SRMR), root mean square error of approximation (RMSEA), goodness of fit index, and comparative fit index [41, 42].

The convergent validities between the BSL-23 and other psychological scales (BHS, BIS-11, CTQ-SF, DERS, PBI, PHQ-9, RSES and UCLA-LS) were analyzed. The difference in BSL-23 scores for high-risk adolescents with and without BPD was also tested. Receiver operating characteristic (ROC) curve analysis was performed to evaluate the discriminating power for BPD diagnosis. The area under the curve (AUC) was used as a measure of the overall performance. The optimal cut-off point was calculated according to Youden’s index, where sensitivity and specificity are valued equally [43].

In addition, the dataset was randomly split into training data (80%) and testing data (20%), and k-fold cross-validation (k = 10) was performed within the training data to obtain the parameters, which were then applied to the testing data.

## Results

### Demographic data

**Table 1:** Demographic characteristics and clinical data

	BPD		no BPD		p value
	N	%	N	%	
Number	157	56.3	122	43.7	
Female	107	87.7	93	59.2	< 0.001
Family history of psychiatric illness	56	46.3	86	54.8	0.160
	**Mean**	**SD**	**Mean**	**SD**	
Age	15.9	1.6	15.3	1.9	< 0.01
Years of education					
Adolescent	9.5	1.6	9.0	1.9	< 0.05
Father	13.3	3.1	13.7	3.3	0.330
Mother	13.2	2.6	13.5	2.7	0.295
Scores of the scales					
BSL-23 (total score)	67.7	18.5	36.3	21.3	< 0.001
BSL-23 (mean score)	2.94	1.24	1.58	1.37	< 0.001
BHS	14.8	4.5	10.9	5.6	< 0.001
BIS-11	67.8	10.8	64.3	10.6	< 0.01
CTQ-SF	54.1	15.1	44.3	11.5	< 0.001
DERS	127.7	20.5	102.7	22.5	< 0.001
PBI					
Paternal care	17.7	7.9	20.1	7.7	< 0.05
Paternal protection	12.4	7.1	11.7	6.4	0.414
Maternal care	18.8	7.8	23.2	7.4	< 0.001
Maternal protection	14.6	7.4	12.7	6.3	< 0.05
PHQ-9	20.3	5.4	13.2	6.4	< 0.001
RSES	17.2	5.1	22.0	6.1	< 0.001
UCLA-LS	31.2	5.3	24.5	6.8	< 0.001

BIS-11: Barratt Impulsiveness Scale, 11th version; BHS: Beck Hopelessness Scale; CTQ-SF: Childhood Trauma Questionnaire, short form; DERS: Difficulties in Emotional Regulation Scale; PBI: Parental Bonding Instrument; PHQ-9: Patient Health Questionnaire-9 item; RSES: Rosenberg Self-Esteem Scale; UCLA-LS: UCLA Loneliness Scale

The demographic characteristics and clinical data of the 279 BPD patients (200 [71.7%] girls, and 79 [28.3%] boys) are shown in Table 1. Their years of age ranged from 12 to 18 years, with a mean of 15.6 years (SD = 1.8). The mean number of years of education of the subjects, their fathers and mothers were 9.2 (SD = 1.8), 13.5 (SD = 3.2) and 13.4 (SD = 2.7), respectively. Half of the patients had a family history of psychiatric disorders.

### Internal consistency and reliability

In our sample (N = 279, BPD = 122), the global Cronbach’s alpha was 0.96, and with the Guttman split-half method the reliability co-efficient was 0.96. The results indicated that the Chinese Mandarin version of the BSL-23 had high internal consistency among high-risk adolescents.

To study the test-retest reliability of the Chinese Mandarin version of the BSL-23, a sub-sample of 32 patients with SITB were asked to complete the instrument again after one week. The results revealed a high correlation (r = 0.936; p < 0.001) between the first (mean = 52.2; SD = 25.0) and second time (mean = 50.4; SD = 26.7) the scale was completed, suggesting high test-retest reliability.

### Factor structure

**Fig. 1:**
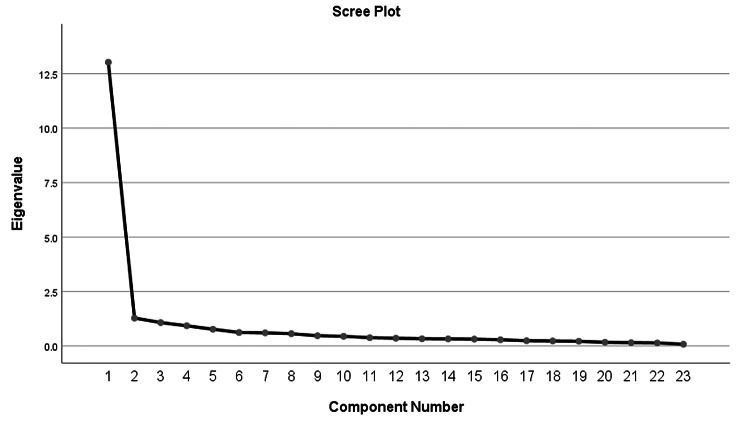
Scree plot of the Chinese Mandarin BSL-23

**Table 2:** Factor loadings (with a varimax rotation) of the Chinese Mandarin BSL-23

BSL-1	0.81	BSL-13	0.75
BSL-2	0.50	BSL-14	0.56
BSL-3	0.76	BSL-15	0.42
BSL-4	0.69	BSL-16	0.80
BSL-5	0.78	BSL-17	0.71
BSL-6	0.54	BSL-18	0.76
BSL-7	0.83	BSL-19	0.59
BSL-8	0.53	BSL-20	0.58
BSL-9	0.64	BSL-21	0.81
BSL-10	0.47	BSL-22	0.52
BSL-11	0.84	BSL-23	0.80
BSL-12	0.75		

The Kaiser-Meyer-Olkin measure of the sampling adequacy of our data was very high (0.958), and Bartlett’s test of sphericity (5326.7) was highly significant (p < 0.001). Both measures indicated that the factor analysis was appropriate for our data. In our data, a single factor explained 56.6% of variance. Although the exploratory factorial analysis showed three factors with eigenvalues greater than 1.0 (13.024, 1.279 and 1.075), cumulatively accounting for 66.9% of the variance, the scree plot (Fig. 1) indicated a one-factor solution. All items showed factorial loadings equal or superior to 0.42, which is an acceptable level for a central factor (Table 2). The goodness of fit test was good (chi square = 977.26, df = 230, p < 0.001). The values of RMSEA, SRMR, comparative fit index, goodness of fit index were 0.099, 0.113, 0.881 and 0.751, respectively.

### Convergent validity

**Table 3:** Correlations between the Chinese Mandarin BSL-23 and other dimensions

Scales	Pearson’s r	p value
BHS	0.597	< 0.001
BIS-11	0.249	< 0.001
CTQ-SF		
Total score	0.423	< 0.001
Physical abuse	0.210	< 0.001
Emotional abuse	0.502	< 0.001
Sexual abuse	0.231	< 0.001
Emotional neglect	0.276	< 0.001
Physical neglect	0.207	< 0.001
DERS	0.806	< 0.001
PBI		
Paternal care	-0.262	< 0.001
Paternal protection	0.131	< 0.05
Maternal care	-0.41	< 0.001
Maternal protection	0.178	< 0.05
PHQ-9	0.807	< 0.001
RSES	-0.662	< 0.001
UCLA-LS	0.639	< 0.001

BIS-11: Barratt Impulsiveness Scale, 11th version; BHS: Beck Hopelessness Scale; CTQ-SF: Childhood Trauma Questionnaire, short form; DERS: Difficulties in Emotional Regulation Scale; PBI: Parental Bonding Instrument; PHQ-9: Patient Health Questionnaire-9 item; RSES: Rosenberg Self-Esteem Scale; UCLA-LS: UCLA Loneliness Scale

There were significant correlations between the Chinese Mandarin version of the BSL-23 and the BHS, BIS-11, DERS, PHQ-9, RSES, UCLA-LS, CTQ-SF and PBI (Table 3).

The correlations between the Chinese Mandarin version of the BSL-23 and the subscales of the PBI indicated that the BSL-23 scores were higher among the patients with lower parental care and higher parental protection (Table 3).

### Difference between high-risk adolescents with and without BPD diagnosis

**Fig. 2:**
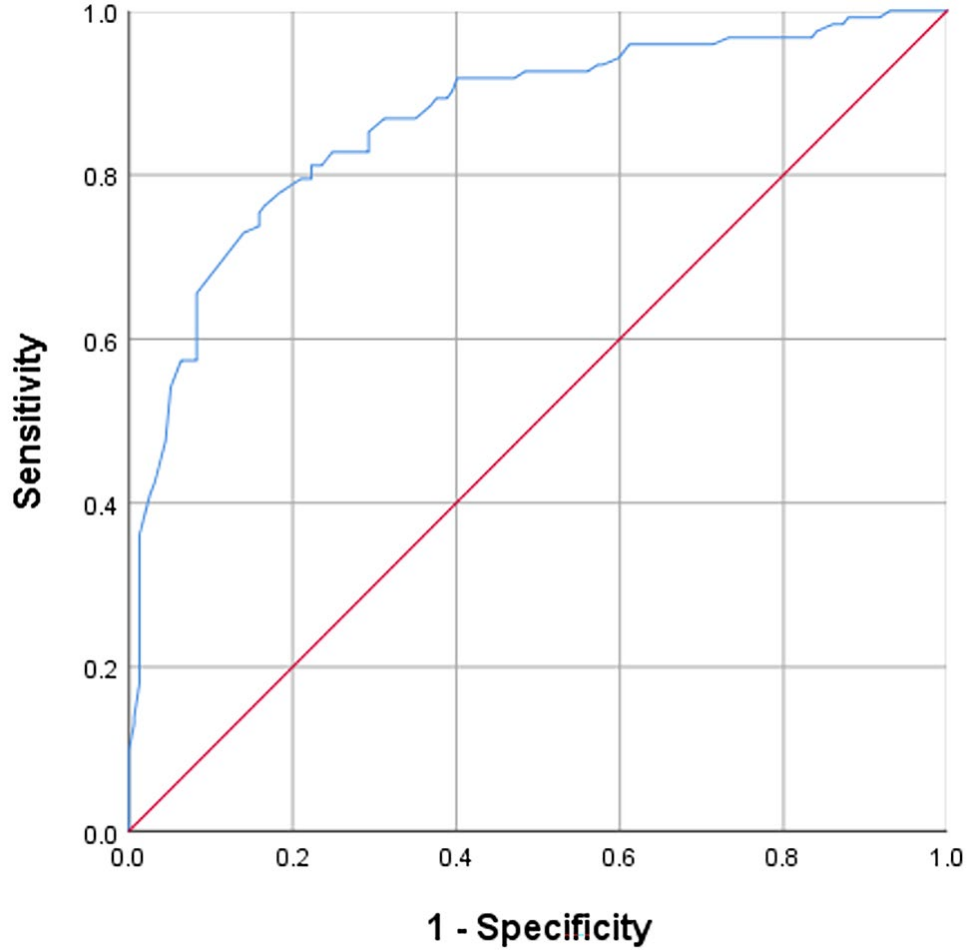
The ROC curve displaying the accuracy of BSL-23 in classifying BPD diagnosis BPD: Borderline personality disorder; ROC: Receiver operating characteristic

Independent sample t tests showed that the Chinese Mandarin version of the BSL-23 could discriminate between suicidal adolescents with BPD and those without BPD assessed with the SCID-II (Table 1). Figure 2 presents the ROC curve and AUC for the Chinese Mandarin version of the BSL-23. The AUC for the measure was acceptable at a value of 0.87 (p < 0.001, 95% CI [0.82, 0.91], SE = 0.02). Youden’s index results indicated that the optimal cut-off value was a total score of 60.5 (mean score 2.63) when giving equal significance to both sensitivity and specificity. Of the 119 patients above this cut-off point, 93 had BPD and 26 did not; for the patients below this cut-off point, 29 had BPD and 131 did not. This cut-off point yielded a sensitivity of 0.76 and specificity of 0.83, with a positive predictive value of 0.78, and negative predictive value of 0.82. The estimated sensitivity, specificity, positive predictive value and negative predictive value of the randomly selected testing data through cross-validation were 0.70, 0.77, 0.70, 0.76, respectively.

## Discussion

This study is the first to evaluate the psychometric properties of the Chinese Mandarin version of the BSL-23 scale in high-risk adolescents. The Chinese Mandarin version of the BSL-23 had high internal consistency and test-retest reliability, and factor analysis showed one highly dominant factor. It showed good concurrent validity with hopelessness (BHS), impulsivity (BIS-11), childhood trauma (CTQ-SF), emotional dysregulation (DERS), depression (PHQ-9), low self-esteem (RSES), and loneliness (UCLA-LS). The AUC showed moderate diagnostic accuracy to discriminate high-risk adolescents with BPD from those without BPD.

Our results showed that the psychometric properties and factor structure of the BSL-23 in suicidal adolescents were similar to those of the previous versions in adults [10–13]. Factor analysis of the original and other versions of the BSL-23 has suggested a one-factor structure, and both the principal component analysis and the scree plot of eigenvalues supported the dominance of a single factor [44]. The goodness of fit test was good. Models with two or three factors were examined, but they did not provide a better fit.

The Chinese Mandarin version of the BSL-23 was correlated with various scales assessing a wide range of symptomatology in high-risk adolescents, with the strongest correlations with severity of depression (r = 0.807), which expands on previous findings that BPD in adolescents is strongly comorbid with depressive disorders [45]. In our study, BSL-23 scores were highly correlated with DERS scores (r = 0.806), showing that emotional dysregulation was related to borderline severity in the enrolled adolescents with SITB. This expands on previous findings that emotional dysregulation assessed with the DERS is correlated with BPD features in adults and nonclinical adolescents [46, 47]. In addition, BSL-23 scores were correlated with BIS-11 scores. This is in concordance with the findings of Cardona et al., who reported that BPD adolescents had higher total BIS-11 scores compared with healthy controls [48]. In summary, correlations between BPD symptoms measured by the BSL-23, depression, emotional dysregulation, and impulsivity suggest that suicidal adolescents with BPD have a greater incidence of affective and behavioral symptoms.

Aside from affective and behavioral aspects, borderline pathology also encompasses cognitive features. Our results showed that BSL-23 scores were correlated with BHS scores, and this is the first direct evidence that adolescents with higher borderline features experience higher levels of hopelessness. Previous studies have shown higher levels of hopelessness in adults with BPD than in those without BPD [49]. Horesh et al. demonstrated that BPD adolescents feel as hopeless as depressive adolescents, and since depressive children experience more hopelessness than healthy controls, one can reasonably argue that BPD adolescents experience more hopelessness than the general population [50, 51]. In our study, BSL-23 scores were correlated with RSES scores, which showed that low self-esteem was related to borderline severity in the enrolled adolescents with SITB. This expands on previous findings that BPD assessed with the Borderline Personality Questionnaire was significantly associated with lower self-esteem in adolescents and young adults [52]. Adolescents with low self-esteem have been reported to develop loneliness due to a feeling of rejection [53]. We also found that BSL-23 scores were correlated with UCLA-LS scores, showing that loneliness was related to borderline severity in the enrolled adolescents with SITB. A twin study showed that loneliness from 12 to 18 years of age was correlated with borderline personality traits at around 19 years of age, mainly due to shared genetic factors rather than environmental influences [54].

A variety of adverse childhood experiences have been identified as important antecedents of BPD. In our study, BSL-23 scores were correlated with CTQ-SF total scores and subscales, showing that childhood trauma was related to borderline severity in the enrolled adolescents with SITB. These findings are consistent with prior studies showing that childhood abuse is an important predictor of BPD in adolescence [55], and that BPD adolescents suffer from more severe childhood abuse and neglect than healthy controls [56]. Specifically, the highest associations were found for emotional and sexual abuse [57]. Emotional abuse has been correlated with more BPD criteria than other forms of abuse, suggesting that the former is a core pathology in BPD [58]. Our results also showed that low parental care and high parental control were correlated with BSL-23 scores when paternal and maternal bonding patterns of the PBI were analyzed separately. This is in line with previous studies among adolescents and adults [57–59] that low parental care and parental overprotection may be a general risk factor for various mental disorders, including BPD [59].

A cut-off value for borderline severity has been proposed by Soler et al. [12], however no study has investigated its diagnostic ability among adolescents with SITB. In the present study, the Chinese Mandarin version of the BSL-23 was correlated with BPD diagnosed by the SCID-II, and the scores were significantly greater among the adolescents diagnosed with BPD compared to those without BPD, indicating the criterion-related validity of the Chinese Mandarin version of the BSL-23 in the evaluation of BPD in high-risk adolescents. This is in line with previous studies which showed that BSL-23 could differentiate between patients with BPD patients and those without BPD with other DSM-IV Axis I disorders or healthy controls [10, 11, 13]. In the current study, the BSL-23 had good criterion-related validity and predictive accuracy (AUC = 0.87) at a cut-off point of a total score of 60/61 (mean score 2.60/2.65) [60] among suicidal adolescents with acceptable sensitivity and specificity (0.76 and 0.83, respectively), which were comparable to previous studies. The BSL-23 scores were demonstrated in terms of both total scores and mean scores, as the former were used by some of the researchers [13] while the latter by the others [11, 12]. The Borderline Personality Features Scale for Children and McLean Screening Instrument for Borderline Personality Disorder have been reported to have moderate to high accuracy in discriminating adolescents with BPD from those without BPD, with AUCs ranging from 0.73 to 0.93 [61, 62].

Several limitations should be noted. First, the BSL-23 is a self-reported measure and is obviously dependent on the introspective ability of an individual. Nonetheless, the positive correlation between BSL-23 scores and SCID-II diagnosis suggests that the patients’ own evaluation was coherent with the clinician’s assessment of BPD. Second, sensitivity to change was not assessed in our study. However, previous studies have shown that different language versions of the BSL-23, including the Chinese Mandarin version, are sensitive to change after DBT interventions for 1 to 12 months [11, 12, 22]. Third, higher BSL-23 scores may not directly infer the severity of borderline features in this study since instruments assessing functional outcomes were not applied. However, the BSL-23 has been used to stratify the severity of BPD in previous studies [21], and our study showed that the Chinese Mandarin version of the BSL-23 could effectively differentiate between patients with and without BPD diagnosis. Lastly, our research only applies to clinical high-risk samples, which might affect the factor structure, convergent validity and cut-off value, and future studies involving community samples are warranted.

## Conclusion

Our study assessed the psychometric properties of the Chinese Mandarin version of the BSL-23 in adolescents involved in SITB. Our results not only confirmed its good internal consistency, reliability and one-factor structure, but also demonstrated a cut-off value to differentiate between adolescents with and without BPD among high-risk adolescents. Moreover, this is the first study to assess the predictive accuracy and cut-off value of the BSL-23. Taken together, our findings suggest that the Chinese Mandarin version of the BSL-23 is an efficient instrument to assess BPD symptomatology and severity in adolescents.

## Data Availability

The datasets used and analyzed during the current study are available from the corresponding author on reasonable request.

## Abbreviations

AUC: Area under the curve
BIS-11: Barratt Impulsiveness Scale, 11th version
BHS: Beck Hopelessness Scale
BPD: Borderline personality disorder
BPQ: Borderline Personality Questionnaires
BSL: Borderline Symptom List
BSL-23: Borderline Symptom List, short form
CTQ-SF: Childhood Trauma Questionnaire, short form
CFI: Comparative fit index
CFA: Confirmatory factor analysis
DBT: Dialectical behavior therapy
DIB-R: Diagnostic Interview for Borderlines-Revised
DERS: Difficulties in Emotional Regulation Scale
DSM-5: The fifth edition of Diagnostic and Statistical Manual of Mental Disorders
EFA: Exploratory factorial analysis
GFI: Goodness of fit index
KMO: Kaiser-Meyer-Olkin
K-SADS-E: Kiddie Schedule for Affective Disorder and Schizophrenia-Epidemiological version for School-Age Children
MMH: Mackay Memorial Hospital
PBI: Parental Bonding Instrument
PHQ-9: Patient Health Questionnaire-9 item
ROC: Receiver operating characteristic
RMSEA: Root mean square error of approximation
RSES: Rosenberg Self-Esteem Scale
SITB: Self-injurious thoughts or behaviors
SITBI: Self-Injurious Thoughts and Behaviors Interview
SRMR: Standardized root mean square residual
SCID-II: Structured Clinical Interview for DSM-IV Axis II Personality Disorders
UCLA-LS: UCLA Loneliness Scale
